# The effects of warm weather on children’s outdoor heat stress and physical activity in a preschool yard in Gothenburg, Sweden

**DOI:** 10.1007/s00484-023-02551-y

**Published:** 2023-09-19

**Authors:** Nils Wallenberg, Fredrik Lindberg, Sofia Thorsson, Jonatan Jungmalm, Andreas Fröberg, Anders Raustorp, David Rayner

**Affiliations:** 1 Department of Earth Sciences, University of Gothenburg, Gothenburg, Sweden; 2 Department of Food and Nutrition and Sport Science, University of Gothenburg, Gothenburg, Sweden; 3 Swedish National Data Service, University of Gothenburg, Gothenburg, Sweden

**Keywords:** Outdoor heat stress, Preschool, Children, Weather, Thermal comfort, Physical activity

## Abstract

Hot weather conditions can have negative impacts on the thermal comfort and physical activity of vulnerable groups such as children. The aim of this study is to analyze the effects of warm weather on 5-year-old children’s thermal comfort and physical activity in a preschool yard in Gothenburg, Sweden. In situ measurements were conducted for 1–1.5 h in the early afternoon on 8 days in May, June, and August of 2022. The thermal comfort and physical activity was estimated with GPS-tracks, heart rate monitors, and step counts and compared to observed weather conditions. Results show that physical activity decreases under warmer weather conditions, depicted by a decrease in distance moved, step counts, and highest registered pulse. Moreover, on warm days, the children avoid sunlit areas. For 50% or more of the time spent in sunlit areas, the children are exposed to cautious levels of heat. In shaded areas, on the other hand, the children are less exposed, with five out of 8 days having 50% or more of the time at neutral levels. The study demonstrates the importance of access to shaded areas in preschool yards where children can continue their active play while simultaneously maintaining a safe thermal status.

**Supplementary Information:**

The online version contains supplementary material available at 10.1007/s00484-023-02551-y.

## Introduction

Warm weather can have negative effects on human health and wellbeing, particularly for certain groups, e.g., elderly (Kovats and Hajat 2008) and children (Kovats and Hajat 2008; Xu et al. 2012) that are at higher risk compared to the general adult population. In addition to a limited thermoregulatory ability in both groups (Kovats and Hajat 2008), elderly also is at higher risk because of cardiovascular and respiratory diseases (Oudin Åström et al. 2011), where high age can be seen as a proxy for chronic disease (Oudin Åström et al. 2015). Children, on the other hand, are at higher risk because they have a higher body-surface-area to body-mass ratio (BSA:BM) (Xu et al. 2012; Vanos 2015; Cheng and Brown 2020. See Haylock et al. 1978 for estimations of BSA), lower sweat rate (Falk and Dotan 2008; Shannon et al. 2009), and are less aware of their own thermal status (Sheffield and Landrigan 2011; Xu et al. 2012; Yun et al. 2014; Kim and de Dear 2018) compared to adults. The higher BSA:BM results in higher convective heat exchange loss compared to an adult during dry events (Vanos 2015). When air temperature (*T*_*air*_) is higher than skin temperature, the higher BSA:BM instead results in a heat gain (Vanos 2015). This is detrimental, especially considering that children’s sweat rate is about half of that of adults (Falk and Dotan 2008; Shannon et al. 2009), which means that they have less ability to cool through evaporative heat loss (Vanos 2015; Cheng and Brown 2020). The fact that children are less aware of their own thermal status and therefore not necessarily know when they are under heat stress conditions is likewise a problem (Sheffield and Landrigan 2011; Xu et al. 2012; Yun et al. 2014; Kim and de Dear 2018). Instead, they are dependent on adults that can ensure their thermal status.

In Sweden, 86% of all 1-to-5-year-old children attend preschool (Swedish National Agency for Education 2022), where they spend around 3 h per day outdoors in the preschool yard (Mårtensson 2004). Raustorp et al. (2012) found that preschoolers in Malmö, Sweden, spend around 47% of their time in preschool outdoors. With such a considerable time spent outdoors in the preschool yard, it is imperative to study the potential influence of weather on the wellbeing of children in preschool age. Bäcklin et al. (2021) found that two thirds of preschool yards in Gothenburg, Sweden, are exposed to strong heat stress on hot and clear days as an effect from absence of abundant shade and that strong heat stress has negative effects on the wellbeing and learning of the children. Comparable results were concluded from a study on preschoolers in Norrköping, Sweden (Malmquist et al. 2021). Moreover, hot weather conditions correlate with increases in emergency department admissions. Bernstein et al. (2022) showed that visits to US children’s hospitals emergency departments increased on days with high outdoor temperatures. In young children aged 0–5, these visits were associated with heat-related illness, infectious or parasitic diseases and ear infections among others. Similar findings are evident for heat-related admissions in 0–14-year-olds in the Netherlands (van Loenhout et al. 2018) and 0–17-year-olds in Ontario, Canada (Wilk et al. 2021).

Physical activity is important for optimal human development and physical and mental health (Bull et al. 2020). Preschoolers over age 5 should accumulate at least 60 min daily physical activity on at least moderate-to-vigorous intensity (MVPA) (Bull et al. 2020). Domains reported to correlate with children’s physical activity are demographic and biological, behavioral, socio-cultural, and physical environment domain (Sallis et al. 2000). The physical environment domain, which includes both the measures of the neighborhood safety and the measures of the preschool physical environment (i.e., play spaces, class size), time spent outdoors, and the specific preschool attended, are correlates reported positively associated with physical activity as well as the quality of the outdoor physical activity environment (Hinkley et al. 2010).

In preschool, children’s time spent in MVPA is reported to predominantly be gathered outdoors (Raustorp et al. 2012). Previous studies from cold (Montreal, Canada (Bélanger et al. 2009) with Dfb Köppen-Geiger climate classification) to temperate climates (Hertfordshire, UK (Goodman et al. 2012) and Auckland, New Zealand (Duncan et al. 2008) with Cfb) have reported a linear relationship of increased temperature associated with increased physical activity. A study by Harrison et al. (2017) reported that within the range of 0–20 °C, this is a reasonable assumption, but that mean daily temperatures below 0 °C showed a flatter relationship with physical activity, and those higher than 20 °C were associated with a decline in physical activity. Ridgers et al. (2015) also observed that girls’ physical activity appeared to be more susceptible to seasonal changes compared to boys, suggesting that strategies to promote physical activity may be needed during the hot summer months, particularly for girls.

The relationship between warm weather and physical activity of small children is relatively unstudied. Most existing research is on older children and adolescents (e.g., Vanos 2015; Vanos et al. 2017; Cheng and Brown 2020; Liu and Jim 2021) and few on smaller children in preschool age (e.g., Xu et al. 2012; Bäcklin et al. 2021; Malmquist et al. 2021; Bernstein et al. 2022; Wallenberg et al. 2023b), where the majority focuses on weather and thermal stress.

One way of addressing the relationship between warm weather and human wellbeing is to use thermal stress indices that describe how different weather conditions are perceived. This can be with a temperature describing the human energy balance (Matzarakis et al. 1999; Blazejczyk et al. 2010) or with the human energy balance directly (Brown and Gillespie 1986; Harlan et al. 2006; Kenny et al. 2009a; Vanos et al. 2017). A drawback with many thermal stress indices is that most are developed for adults, thus omitting the differences between children and adults, e.g., sweat rate and BSA:BM. Vanos et al. (2017) studied the human energy balance of children aged 9–13 during different physical activities using the COMfort FormulA (COMFA) model (Brown and Gillespie 1986). The physical activities were observed using Polar Team Pro that measures heart rate and GPS-tracks (Vanos et al. 2017). The heart rate was utilized to estimate metabolic heat production of the children. COMFA is originally developed for calculations of the human energy balance of adults, but is recently available as COMFA-kid (Cheng and Brown 2020) explicitly for studies on small children, considering the differences in sweat rate and BSA:BM. The advantage with COMFA is that it is responsive to changes in physical activity, which influences metabolic heat production, as compared to other thermal stress indices (Liu and Jim 2021; Wallenberg et al. 2023b).

The aim of this paper is to analyze the outdoor thermal stress and physical activity of 5-year-old children in a preschool yard in Gothenburg, Sweden, based on eight early afternoon sessions in May, June, and August 2022. The state-of-the-art methodology utilizes Global Positioning System (GPS) locations and heart rate of the children that is compared to prevailing weather conditions to investigate if there is an effect of weather on the physical activity and wellbeing.

## Methods

### Study area

**Fig. 1:**
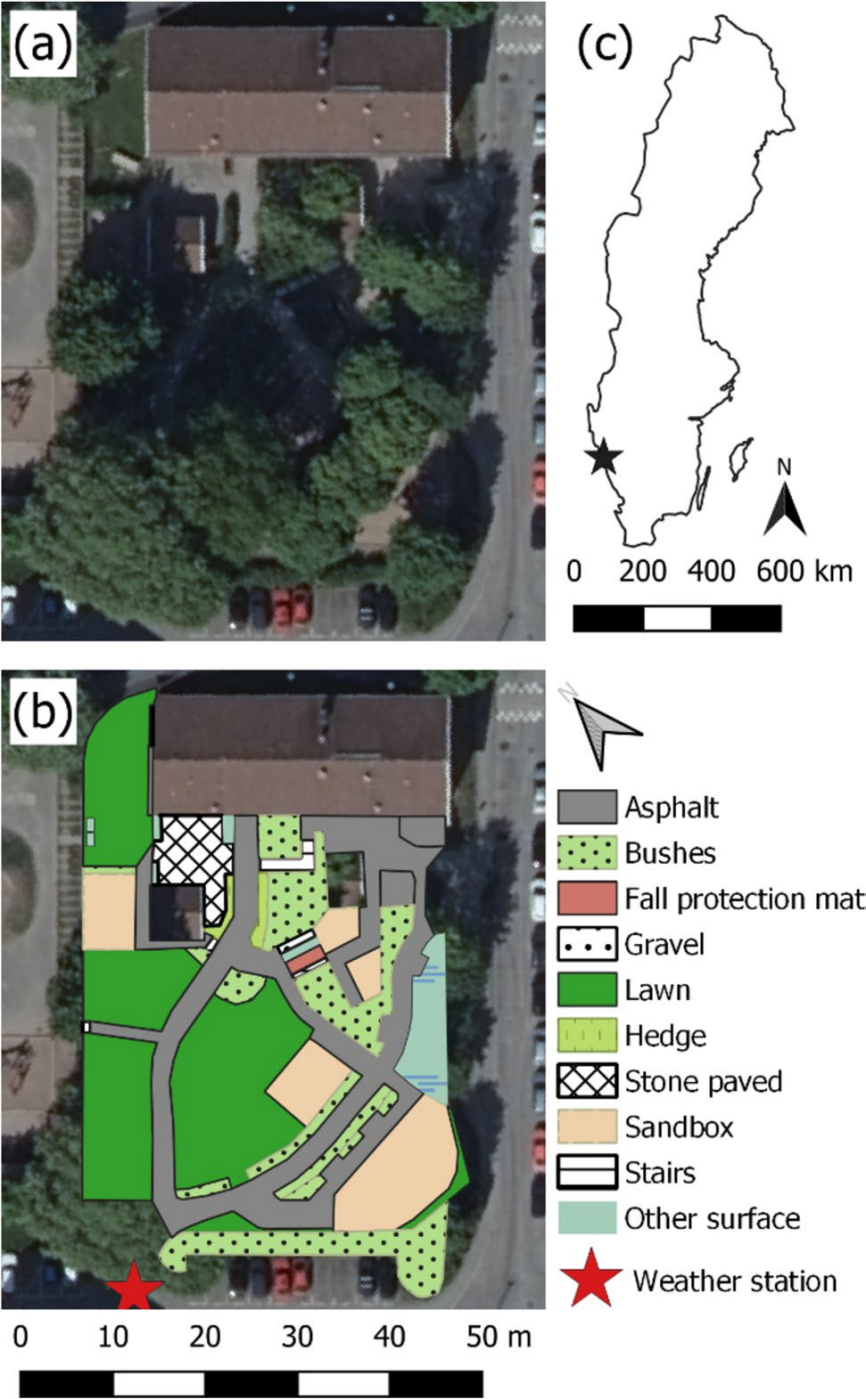
Figure showing **a** ortho photo of the preschool yard and **b** ortho photo overlaid with vector data showing the different areas within the preschool yard area and the location of the weather station, and **c** map of Sweden with location of Gothenburg (black star)

The preschool yard is located in an open midrise local climate zone (Stewart and Oke 2012) in the Sandarna area of Gothenburg. The preschool yard is lush with bushes and large trees (visible in Fig. 1a) providing shade in most of the 1875 m^2^ of the preschool yard on clear days. Much of the yard consists of permeable surfaces (e.g., lawn, sand, and bushes. See Fig. 1b). Part of the preschool yard is hilly (upper right corner in Fig. 1a–b), with a slide and bushes. The location of Gothenburg in Sweden is given in Fig. 1c (57°41′N 11°55′E). The climate in Gothenburg falls within the Dfb category in the Köppen-Geiger climate classification described as a cold climate with mild to cold winters and relatively warm summers (Beck et al. 2018). The daily mean *T*_*air*_ for Gothenburg (1991–2020) in May, June, and August are 12.3, 15.9, and 17.8 °C, respectively, with mean maximum *T*_*air*_ for same months of 24.5, 26.8, and 27.8 °C (SMHI 2022a). Mean wind speeds for May, June, and August are 3.0, 3.1, and 2.6 m/s, respectively, with winds predominantly from the south (SMHI 2022b).

### Measurement and observations of children’s activity level

Nine 5-year-old preschoolers, five girls and four boys (see Table 1 for mean, min, and max weight, height, and BMI), were selected to participate in the study based on consent from their parents. Attendance varied between days, with four children as the lowest number and nine as highest. Collection took place for 1–1.5 h in the early afternoon (1–3 pm) and was repeated for 8 days in May, June, and August of 2022 (see Table 2 for details).

**Table 1:** Mean, min, and max weight (kg), height (cm), BMI, resting heart rate (bpm), and maximum heart rate (bpm) of the five girls and the four boys that participated in the study

	Mean	Min	Max
Girls (*n* = 5)			
Weight (kg)	22.4	18.3	26.6
Height (cm)	115.9	109.7	119.1
BMI	16.6	14.7	19.5
Resting heart rate (bpm)	74	67	83
Maximum heart rate (bpm)	199	194	205
Boys (*n* = 4)			
Weight (kg)	22.3	20.0	24.6
Height (cm)	119.0	114.9	126.0
BMI	15.7	14.5	16.7
Resting heart rate (bpm)	79	69	92
Maximum heart rate (bpm)	196	190	203

**Table 2:** Mean air temperature (*T*_*air*_ (°C)), relative humidity (*RH* (%)), wind speed (*ws* (m/s)), global horizontal shortwave radiation (*K*_↓_ (Wm^−2^)), direct horizontal shortwave radiation (*K*_*I*_ (Wm^−2^)), and diffuse horizontal shortwave radiation (*K*_*D*_ (Wm^−2^)), for each respective date and time span (Time) accompanied by an interpretation of the weather conditions for *T*_*air*_, wind speed, and insolation. Rows are ordered from coolest to warmest day

Time	Weather	*T*_*air*_ (°C)	*RH* (%)	*ws* (m/s)	*K*_↓_ (Wm^−2^)	*K*_*I*_ (Wm^−2^)	*K*_*D*_ (Wm^−2^)
2022-06-01 13:20—14:30	Cool/calm/sunny	15.1	50.5	1.2	771.7	624.6	147.1
2022-05-23 13:30—14:30	Cool/calm/sunny	16.9	40.9	1.5	713.5	499.2	214.3
2022-06-14 13:10—14:20	Cool/calm/sunny	17.9	50.6	1.9	821.7	707.9	113.8
2022-06-27 13:00—14:05	Warm/calm/overcast	20.8	73.8	0.9	181.6	35.3	146.3
2022-06-29 13:05—14:05	Warm/calm/sunny	20.8	54.3	1.8	789.5	647.2	142.3
2022-08-17 13:05—14:25	Warm/calm/overcast	21.9	66.5	0.9	277.0	86.3	190.8
2022-08-18 13:10—14:10	Hot/calm/semi-cloudy	24.3	49.0	1.2	506.8	280.8	226.1
2022-06-30 13:10—14:10	Hot/calm/semi-cloudy	26.4	33.6	1.3	489.4	267.8	221.6

The outdoor activity of the children was examined using Polar Team Pro sensors (Polar, Kempele, Finland). The Polar Team Pro sensors have previously been used to determine thermal comfort of 9–13-year-olds (Vanos et al. 2017). Child heart rate (bpm), speed (m/s), distance (m), and steps (per minute) as well as child location (lat and lon) were measured with a pulse strap including a GPS-tracker, attached to the chest. Ten data points were collected every second (10 Hz) and then converted to 1-s averages. A complimentary gathering of objectively measured physical activity data was made with a research pedometer (Yamax SW 200 (Tokyo Japan)). A comparison (not shown) of steps from the Polar Team Pro and steps from the Yamax SW 200 showed high agreement (*R*^2^ = 0.84). The main reason for using two devices was to facilitate direct comparison of child activity to previously conducted research. GPS points on top of buildings or outside the preschool yard perimeter were removed, i.e., where the ortho photo is visible in Fig. 1b. Observations where heart rate was below resting heart rate (e.g., zero, because the sensor was recording but not attached) were also removed, as well as observations that differed with >5 min from weather data (only relevant in the beginning and end of observations).

### SOlar and LongWave Environmental Irradiance Model (SOLWEIG)

The SOlar and LongWave Environmental Irradiance Model (SOLWEIG) (Lindberg et al. 2008) was used to calculate short- and longwave fluxes as well as shadow patterns on the preschool yard. SOLWEIG has been used and evaluated in numerous studies on thermal stress of humans in outdoor environments (e.g., Lindberg et al. 2016; Thom et al. 2016; Thorsson et al. 2017; Bäcklin et al. 2021). Required input are meteorological data (global shortwave radiation, *T*_*air*_, and relative humidity) and a digital surface model (DSM) with information on building elevation. Optional input data are a canopy digital surface model (CDSM) with vegetation height, ground cover data with information on emissivity and albedo of different surfaces, and more detailed meteorological data (e.g., direct and diffuse shortwave radiation). Here, 0.5-m spatial resolution DSM, CDSM, and ground cover data for the preschool yard together with observed weather conditions (direct and diffuse shortwave radiation, *T*_*air*_, relative humidity, and air pressure; see “Observed meteorological variables” section) was used as input data into SOLWEIG. The anisotropic radiation schemes described in Wallenberg et al. (2020, 2023a) were utilized. Radiant load, here described with *R*_*abs*_ (Wm^−2^), is estimated in SOLWEIG from simulated short- and longwave fluxes and used as input into the COMFA model that estimates the human energy balance (see “COMfort FormulA model” section). In addition, SOLWEIG is utilized to estimate shadow patterns, which are used to together with the GPS-tracks to determine if the children are in sunlit or shaded parts of the preschool yard.

### Observed meteorological variables

**Fig. 2:**
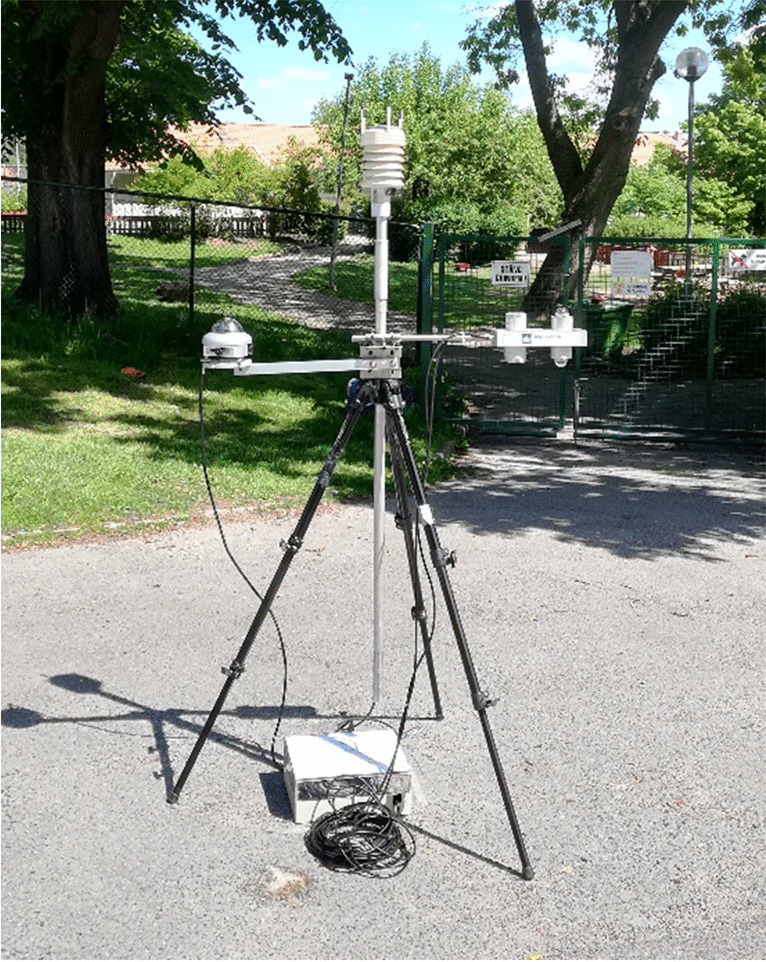
Weather station with a Vaisala Weather Transmitter WXT520, Delta-T SPN1 Sunshine Pyranometer, and Kipp & Zonen CNR1 Net Radiometer connected to a Campbell Scientific CR1000 logger

The preschool yard’s microclimate was measured with a stationary weather station (Fig. 2) just outside the preschool yard’s perimeter to avoid the children accidentally interfering with it. *T*_*air*_, relative humidity, and wind speed were measured with a Vaisala Weather Transmitter WXT 520 (Vaisala 2012). Global (*K*_↓_) and diffuse (*K*_*D*_) horizontal shortwave radiation was measured with a Delta-T SPN1 Sunshine Pyranometer (Wood 2019). Direct horizontal shortwave radiation (*K*_*I*_) is estimated from *K*_↓_ and *K*_*D*_. The instruments were mounted on a tripod ~1.1 m above ground. Observations were made every 5 s and averaged over every 5 min. Detailed information on, e.g., response times, measuring ranges, instrument uncertainties, and calibration, can be found in respective instrument reference.

Table 2 shows mean meteorological data for each day and time when the children were outside. Lowest mean *T*_*air*_ (15.1 °C) was recorded on 2022-06-01 and highest mean *T*_*air*_ (26.4 °C) was observed on 2022-06-30. Cloudiness varied between days, evident in *K*_↓_, *K*_*I*_, and *K*_*D*_, with lowest mean *K*_↓_ on 2022-06-27 of 181.6 Wm^−2^ (*K*_*I*_ = 35.3 Wm^−2^) corresponding to cloudy/overcast conditions, whereas 2022-06-14 had a mean *K*_↓_ of 821.7 Wm^−2^ (*K*_*I*_ = 707.9 Wm^−2^) typical for a cloudless day in extratropical latitudes. None of the days had notably high wind speeds. All variables except *K*_↓_ were used as input to SOLWEIG (see “SOlar and LongWave Environmental Irradiance Model (SOLWEIG)” section).

### COMfort FormulA model

The COMFA thermal comfort model (Brown and Gillespie 1986; Kenny et al. 2009a, 2009b; Vanos et al. 2012a, 2012b) is an increasingly popular model that can be used for thermal comfort assessments and heat stress studies (Vanos et al. 2012c; Vanos et al. 2017; Liu and Jim 2021; Wallenberg et al. 2023b). In short, the COMFA model estimates the energy balance (*EB*) (Wm^−2^) of a human as follows:

1 $$EB=M+{R}_{abs}-E-C-{L}_{emit}$$

where *M* is the metabolic heat production, *R*_*abs*_ is the radiant load (absorbed short- and longwave radiation), *E* is the evaporative heat loss, *C* is the convective heat loss, and *L*_*emit*_ is the longwave emitted heat loss. Radiant load (*R*_*abs*_) is calculated based on short- and longwave radiation fluxes from the surroundings. These fluxes were estimated in SOLWEIG (“SOlar and LongWave Environmental Irradiance Model (SOLWEIG)” section) based on meteorological input data from the weather station (“Observed meteorological variables” section). Metabolic heat production (*M*) is estimated based on age, gender, weight, height, and activity, where age, gender, weight, and height are known and fixed, whereas activity differs depending on heart rate. Therefore, metabolic heat production differs depending on activity, i.e., metabolic equivalent task (MET). Standing still and running would, for example, have contrasting MET. Here, COMFA has an advantage in that these differences are apparent in the resulting metabolic heat production and energy balance (e.g., Vanos et al. 2012c), which is not the case with many other popular thermal comfort indices. The original COMFA model is developed for studies on adults, as is the case for most thermal comfort indices. Cheng and Brown (2020), on the other hand, modified the original COMFA model to be used on children (COMFA-kid), by changing the resting metabolic rate, sweat rate, and body-surface area to body-mass ratio. In this study, we use COMFA-kid.

MET is estimated based on the activity heart rate (*AHR*) of the preschooler, i.e., for every second, following the methods by Strath et al. (2000). In their method, estimated heart rate reserve (*HHR*) (%) is equal to percentage of maximum oxygen consumption (*VO*_2*reserve*_) and is estimated from the *AHR*, resting heart rate (*RHR*), and maximum heart rate (*MHR*) according to Eq. 2:

2 $$HHR=\frac{AHR- RHR}{MHR- RHR}\times 100$$

With *VO*_2*reserve*_ equal to *HHR*, it is possible to calculate activity oxygen consumption (*VO*_2*act*_) from the following equation:

3 $${VO}_{2 reserve}=\frac{VO_{2 activity}-{VO}_{2 resting}}{VO_{2\mathit{\max}}-{VO}_{2 resting}}\times 100$$

where resting oxygen consumption (*VO*_2*resting*_) is 3.5 mL kg^−1^ min^−1^ and maximum oxygen consumption (*VO*_2*max*_) is estimated according to Jackson et al. (1990):

4 $${VO}_{2\max}=50.513+1.589\left(PA\left[0-7\right]\right)-0.289(years)-\;0.552\left(\%fat\right)+5.863\left(F=0,M=1\right)$$

In Eq. 4, *PA* is physical activity status, *years* is the age in years of the preschooler, %*fat* is percent body fat, and *F* = female and *M* = male. In this study, physical activity status is set to 4 (healthy physically active children, based on author’s opinion) and percent body fat is based on average for 5-year-old girls (21%) and boys (17%) from Karlsson et al. (2013). MET for the present activity (heartbeat) is estimated from *VO*_2*activity*_ by dividing with *VO*_2*resting*_, i.e., 3.5 (value for adults) according to Strath et al. (2000). Metabolic activity (*M*_*act*_) is used as input into the COMFA model to calculate metabolic heat. *M*_*act*_ is calculated from MET and resting metabolic rate (RMR), where RMR is estimated for 3–10-year-old girls and boys according to Schofield (1985).

The output energy balance from COMFA is compared to the 7-point scale for non-exercising activities by Kenny et al. (2009b). This scale can be compared to the thresholds for heat stress proposed by Harlan et al. (2006), where caution should be paid to prolonged exposure to energy balances ranging 65–120 Wm^−2^, which can lead to fatigue and discomfort. Extreme caution should be taken to energy balances ranging 121–200 Wm^−2^, which can lead to sunstroke, heat cramps, and heat exhaustion. Continuous exposure to energy balances exceeding 200 Wm^−2^ is considered dangerous, with previously mentioned symptoms likely and heatstroke possible. It is considered extremely dangerous when the energy balance is above 340 Wm^−2^ with sunstroke and heatstroke as likely outcomes. The “danger” threshold (200 Wm^−2^) is, however, set according to apparent temperature (40 °C) that residents in Phoenix, USA, are accustomed to (Harlan et al. 2006), which is rarely if ever reached in Sweden. Thus, this threshold is potentially lower than 200 Wm^−2^ for persons acclimatized to Swedish conditions.

## Results

**Fig. 3:**
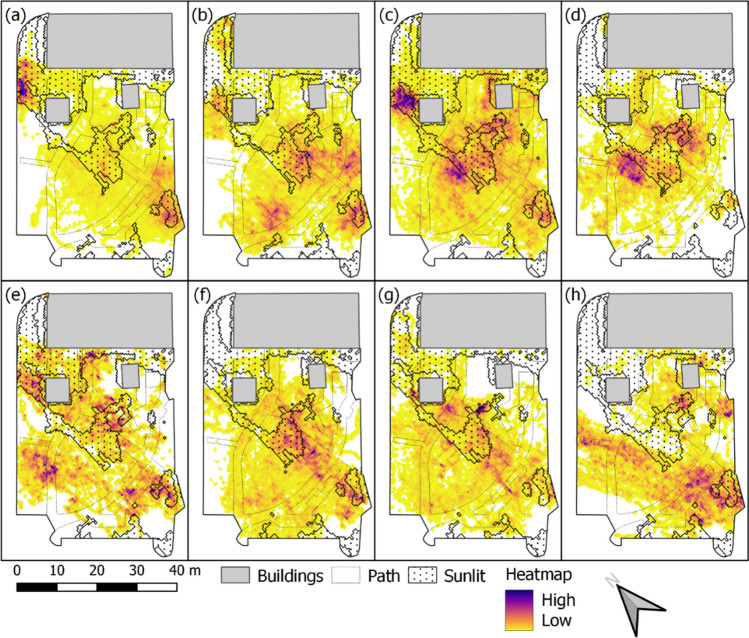
Kernel density maps showing areas frequented by the children for **a** 2022-06-01, **b** 2022-05-23, **c** 2022-06-14, **d** 2022-06-27, **e** 2022-06-29, **f** 2022-08-17, **g** 2022-08-18, and **h** 2022-06-30. High indicates areas that are frequented intensively and low are areas that are frequented less. Dotted areas are sunlit. Subplots are ordered from coolest to warmest day (*T*_*air*_, see Table 2)

Kernel density maps, ordered from coolest to warmest day (*T*_*air*_), showing where and how frequently children have been in certain areas of the preschool yard on each individual day are given in Fig. 3. Dots indicate that the area was sunlit in all model time steps. The remaining areas had shade in one or more model time steps. Most striking here is that on the warmest day (Fig. 3h) children avoided sunlit areas (dotted). On the three coolest days (Fig. 3a–c), a hot spot in the upper left corner is visible, where some of the children were seated at a table and painted. This table was later moved into shade.

The shadow patterns estimated in SOLWEIG are used to determine if the children are in shaded or sunlit locations. From this, time spent in shadow (%) and time spent in sun (%) were calculated, based on all GPS points for each individual day (i.e., all children). The results are given in Table 3. Time spent in sunlit areas ranges from 13 to 39%, where largest amount of time (39%) is on the coolest day (2022-06-01) and lowest (13%) on the warmest day (2022-06-30).

**Table 3:** Percentage of the preschool yard area used by the GPS-tracked children and their corresponding time spent in shade and sun. Rows are ordered from coolest to warmest day (*T*_*air*_, see Table 2)

Date	Area used (%)	Time spent in shadow (%)	Time spent in sun (%)
2022-06-01	67	61	39
2022-05-23	73	77	23
2022-06-14	86	69	31
2022-06-27	59	75	25
2022-06-29	64	72	28
2022-08-17	68	78	22
2022-08-18	70	79	21
2022-06-30	61	87	13

**Fig. 4:**
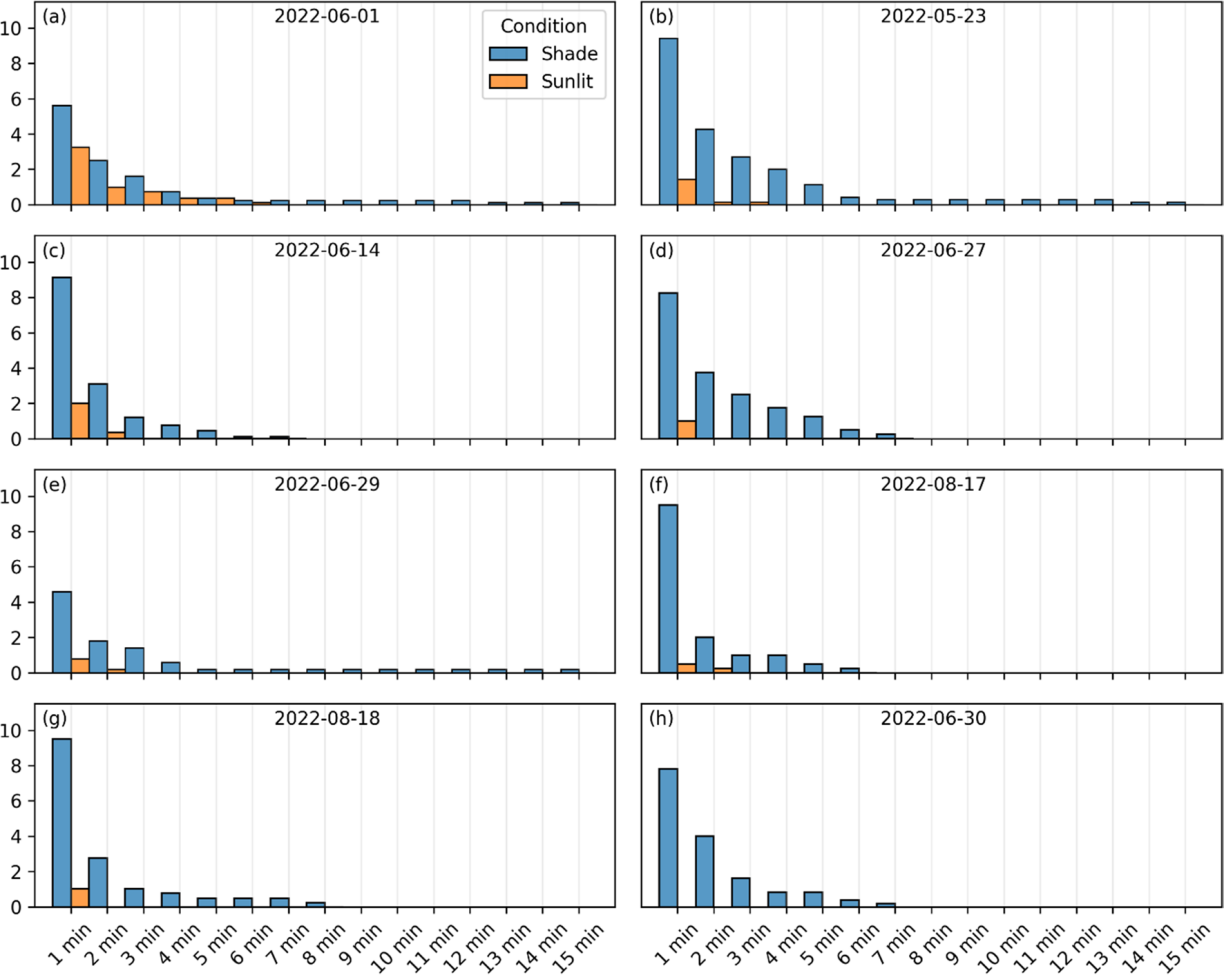
Average number of times a child has spent minimum 1 min up to 15 min continuous time in sun (orange) and shade (blue) for **a** 2022-06-01, **b** 2022-05-23, **c** 2022-06-14, **d** 2022-06-27, **e** 2022-06-29, **f** 2022-08-17, **g** 2022-08-18, and **h** 2022-06-30. Numbers are cumulative, i.e., if a child spent 2 min continuous time in sun it is also counted in 1 min. Subplots are ordered from coolest to warmest day (*T*_*air*_, see Table 2)

Estimations of continuous time spent in shaded and sunlit areas differed between days (Fig. 4). On the coolest day, children spent up to six continuous minutes in sunlit areas without moving into shade (Fig. 4a). This can be compared with the warmest day where the observed children never reached one continuous minute in sunlit conditions (Fig. 4h). The children were allowed to spend <5 s in the opposite area (sunlit if looking at time spent in shade and vice versa) as to not remove tracks when passing through, e.g., a sunlit area. Thus, calculations of continuous time spent in sunlit or shaded areas can include instances <5 s in the opposite area.

**Fig. 5:**
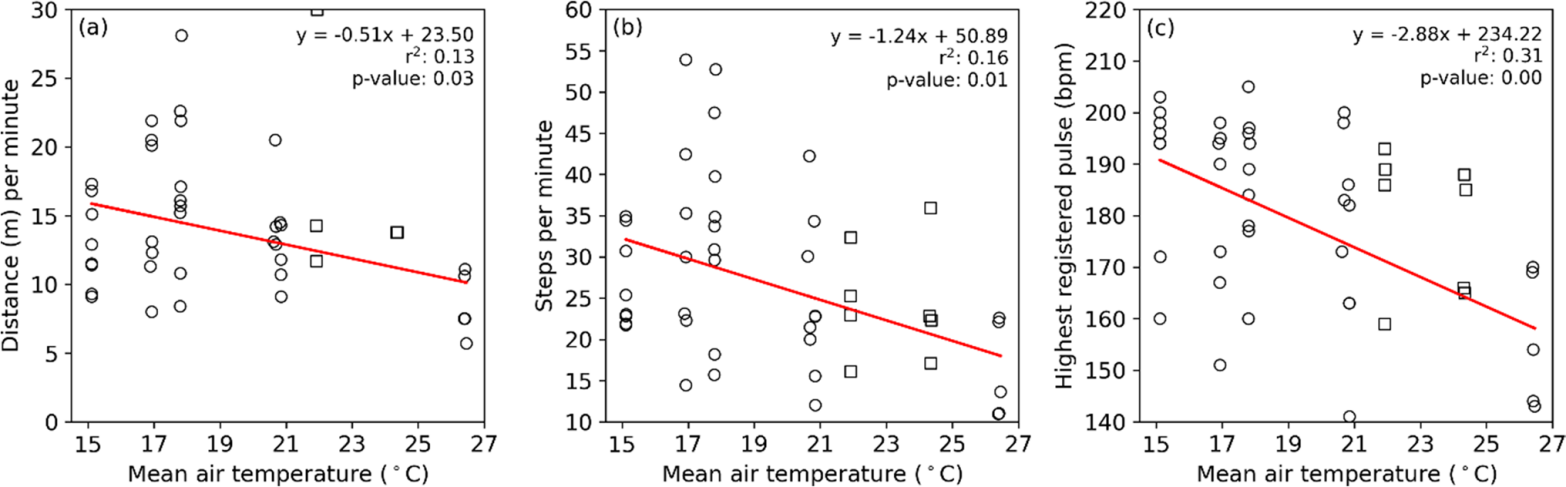
Scatter plots between mean air temperature and **a** distance per minute, **b** steps per minute, and **c** highest registered pulse, with corresponding slope, intercept, *r*^2^, and *p* value. Data points depicted with circles are included in the regression analysis. Data points illustrated with squares are excluded from the regression analysis because the children were riding bikes, compared to the other days when they were on foot

Mean *T*_*air*_ can explain some of the behaviors of the preschoolers. In Fig. 5a, a scatter plot between mean *T*_*air*_ and distance per minute (m) is given and shows that distance moved per minute decreases with an increase in *T*_*air*_ (*r*^2^ = 0.13). Steps per minute, likewise, decreases with an increase in *T*_*air*_ (Fig. 5b). This indicates that as *T*_*air*_ increases, physical activity decreases. This is also evident in the highest registered pulse (Fig. 5c), where the highest registered pulse decreases with an increase in *T*_*air*_. Here, 31% of the variance is explained by *T*_*air*_. The last 2 days (2022-08-17 and 2022-08-18) were left out of this analysis as the children were on bikes, affecting the distance and steps per minute, compared to the other days when they were on foot.

**Fig. 6:**
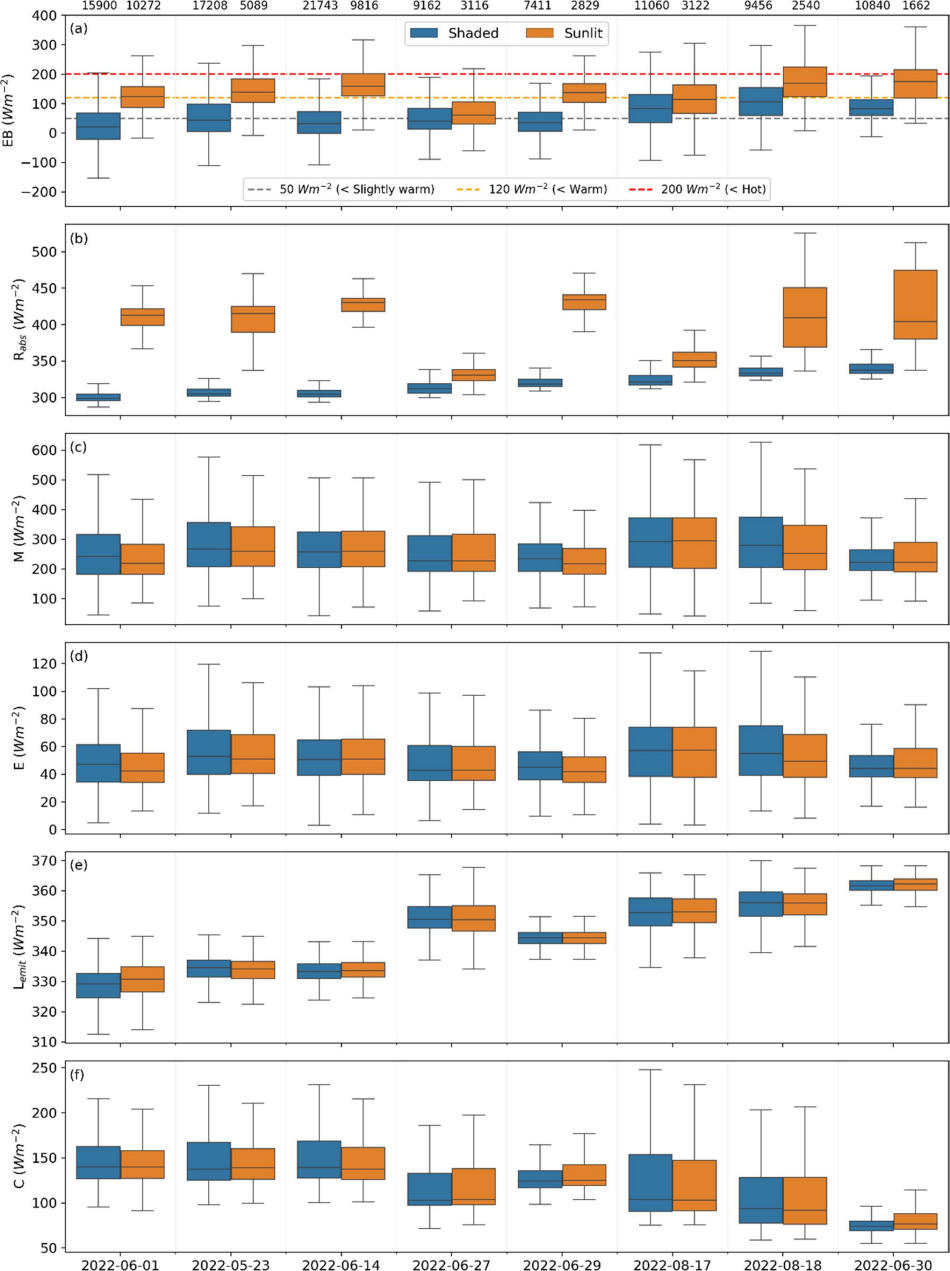
Box plots showing the distribution (minimum, first quartile, median, third quartile, and maximum) of **a** the energy balance (*EB*), **b** radiant load (*R*_*abs*_), **c** metabolic heat production (*M*), **d** evaporative heat loss (*E*), **e** emitted longwave heat loss (*L*_*emit*_), and **f** convective heat loss (*C*) for each day in sunlit (orange) and shaded (blue) conditions. The numbers above **a** are the number of observations in each group (shaded and sunlit) and are equal for all variables for the corresponding day. The data in each subplot is ordered from coolest to warmest day (*T*_*air*_, see Table 2)

Results from calculations of the energy balance with COMFA and the individual variables (*R*_*abs*_, *M*, *E*, *L*_*emit*_, and *C*), divided into shaded and sunlit conditions, are presented in Fig. 6. Figure 6a shows the estimated energy balance from COMFA each day. The energy balance is logically always higher in sunlit conditions compared to shaded conditions. Two days, 2022-06-27 and 2022-08-17, show minor differences between sunlit and shaded conditions. These 2 days had the lowest average *K*_↓_, indicating that they are overcast/cloudy, resulting in small differences between what would otherwise be sunlit and shaded conditions. Moreover, it is evident that the energy balance for 50% or more of the time spent in sunlit locations exceeds 120 Wm^−2^, i.e., warm conditions, on all days except 2022-06-27 and 2022-08-17. In shaded locations, on the other hand, around 75% or more of the time on all days except one (2022-08-17) the energy balance of the children is estimated to be below 120 Wm^−2^ (warm), and below 50 Wm^−2^ (slightly warm) on all days except the last three.

The differences in energy balance between sunlit and shaded conditions can be attributed to radiant load. Radiant load (*R*_*abs*_) is given in Fig. 6b. Here, substantial differences are noticeable between shaded and sunlit points as an effect of exposure to shortwave (solar) radiation in the sunlit locations. These differences indicate that radiant load is the most influential variable for the resulting estimations of the energy balance seen in Fig. 6a, explaining the variations between shaded and sunlit conditions. Here, only 2 days have minor differences between sunlit and shaded conditions, same days as described above (2022-06-27 and 2022-08-17), explained by the prevailing overcast/cloudy conditions on these 2 days.

Metabolic heat production (*M*), given in Fig. 6c, shows minor differences in median, regardless of day and condition (sunlit or shaded). Largest range in metabolic heat production is visible on the last 2 days of measurements, 2022-08-17 and 2022-08-18. On these 2 days, the kids were on and off riding bikes.

Evaporative heat loss (*E*) through perspiration, shown in Fig. 6d, is similar in median for all days. The largest ranges are visible on the last 2 days.

Emitted longwave radiation (*L*_*emit*_) (Fig. 6e) corresponds to average *T*_*air*_ for each day, where 2022-06-30 is the hottest day, resulting in the highest median emitted longwave radiation. The lowest median emitted longwave radiation, on the other hand, is on 2022-06-01, which was also the coolest day.

Convective heat loss (*C*), in Fig. 6f, is similar on all days (small range). The values are influenced by *T*_*air*_, wind speed, and skin temperature, where wind would remove energy from the body (increase convective heat loss). If skin temperature, on the other hand, is close to *T*_*air*_ convective heat loss will decrease. Wind speed is similar and low on all days (Table 2). The difference between average *T*_*air*_ and average skin temperature (not shown) is, however, smallest on 2022-06-30, explaining why convective heat loss is lowest on this day.

**Fig. 7:**
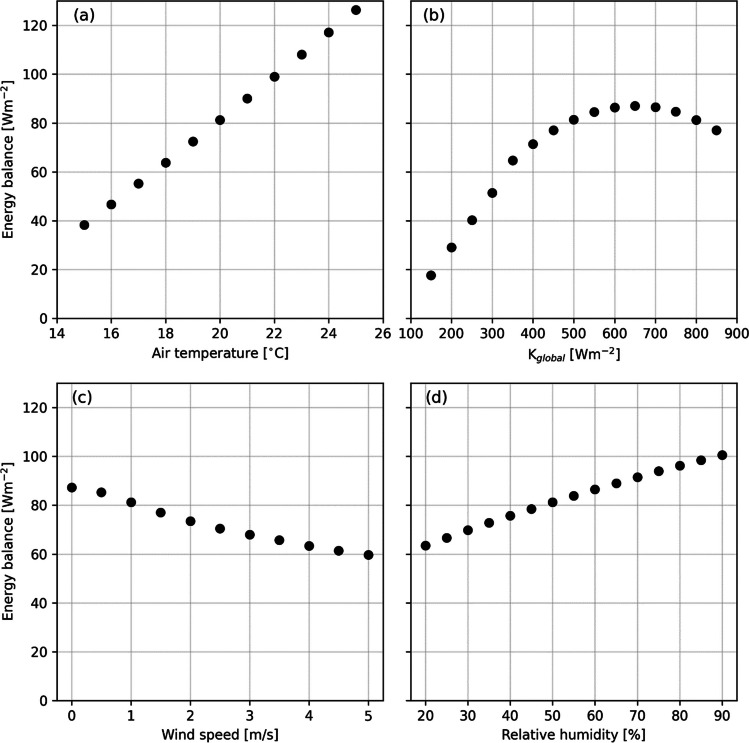
Scatter plots showing the resulting energy balance for different inputs of **a** *T*_*air*_ (°C), **b** *K*_↓_ (Wm^−2^), **c** wind speed (m/s), and **d** relative humidity (%) for a 5-year-old boy with a weight of 22.3 kg and height of 119 cm. The resting and maximum heart rates are set to 79 and 196, respectively, with an activity heart rate set to 100, estimating to a MET of ~3.5. A fixed *K*_↓_ of 800 Wm^−2^ is used in **a**, **c**, and **d**. *T*_*air*_ is set to 20 °C in **b**–**d**; wind speed to 1.0 m/s in **a**, **b**, and **d**; and relative humidity to 50% in **a**–**c**. Calculations are for solar altitude and azimuth in Gothenburg, Sweden, on 2022-06-30 14:00 LST

In Fig. 7, sensitivity tests on the influences of the four input meteorological variables to COMFA, *T*_*air*_, *K*_↓_, wind speed, and relative humidity, on the resulting energy balance of a 5-year-old boy (22.3 kg and 119 cm height) are shown. *T*_*air*_ (Fig. 7a) and *K*_↓_ (Fig. 7b) have the largest effects on the energy balance, whereas the effects of wind speed (Fig. 7c) and relative humidity (Fig. 7d) are small in comparison. The model by Reindl et al. (1990) was used in the sensitivity tests to calculate *K*_*I*_ and *K*_*D*_ from *K*_↓_. This results in a peak in energy balance when *K*_↓_ is around 650 Wm^−2^ as an effect of a combined high exposure to *K*_*I*_ and *K*_*D*_. *K*_↓_ and wind speed are two variables that can differ considerably on a spatial scale, where the differences in *K*_↓_ depend mainly on shadow patterns, which are considered here. Wind speed, on the other hand, is influenced by surrounding obstacle geometries, e.g., buildings and trees. In this study wind speed is the same for the entire preschool yard, based on the measured wind speed outside the preschool yard. Although it is a limitation using the same wind speed for the entire yard, the author’s consider wind speed being representative, seeing that wind speed is low on all days (mean < 2 m/s at 2 m height). Therefore, major differences in energy balance are not likely.

## Discussion

The results presented here show that weather influences the heat stress and physical activity of the children in a preschool in Gothenburg, Sweden. The children seek shade on warm days to alleviate heat gain and increase their thermal comfort. Moreover, their physical activity decreases as the weather becomes warmer, indicated by shorter distances moved, fewer steps taken, and a reduction in highest registered pulse compared to cooler days (Fig. 5). Thirty-one percent of the variance in highest registered pulse is explained by *T*_*air*_. Previous research on the effect of weather on Swedish preschoolers have shown that many preschool yards are exposed to strong heat stress during warm and clear weather conditions (Bäcklin et al. 2021). Heat stress has negative effects on the pedagogical activities and wellbeing of the children (Bäcklin et al. 2021; Malmquist et al. 2021). Nevertheless, little is known about the effect of warm weather on outdoor physical activities of children in preschool yards. Bäcklin et al. (2021) proposed in situ research with GPS-trackers and heart rate monitors to investigate how children’s activity and wellbeing are affected by weather. The results presented here together with the findings by Bäcklin et al. (2021) and Malmquist et al. (2021) show that warm weather conditions have negative effects on the outdoor physical activities as well as the pedagogical activities and wellbeing of Swedish preschoolers.

The simulated energy balances by the COMFA model (Fig. 6) shows that exposure to heat is lower in shaded areas on all days, in agreement with the study on 9–13-year-olds by Vanos et al. (2017). On all days except one (2022-06-27, which was cloudy/overcast and had low *T*_*air*_), the children’s median energy balance surpassed 120 Wm^−2^, i.e., warm conditions (Vanos et al. 2017) in sunlit areas. Prolonged exposure to energy balances above 120 Wm^−2^ can lead to sunstroke, heat cramps, and heat exhaustion and extreme caution is advised (Harlan et al. 2006). In shaded areas, on the other hand, median energy balances never surpassed 120 Wm^−2^ and were below 50 Wm^−2^ (slightly warm) on all days except the three hottest days. The sensitivity test shows that shortwave radiation and *T*_*air*_ have largest effect on the energy balance. While *T*_*air*_ is difficult to influence, direct shortwave radiation can be blocked by trees (Lee et al. 2013; Konarska et al. 2014; Middel et al. 2021) and buildings (Lee et al. 2013). This emphasizes the importance of abundant shade in preschool yards, where the children can escape the direct sunlight. The shade in the preschool yard in this study is primarily by trees and as indicated the trees are highly efficient in lowering radiant load and increasing thermal comfort on hot and sunny days, in line with previous research (Shashua-Bar et al. 2009; Lee et al. 2016; Bäcklin et al. 2021; Middel et al. 2021; Wallenberg et al. 2023b). Shading can likewise mitigate high surface temperatures (Vanos 2015; Middel et al. 2021) in favorable areas of the preschool yard, e.g., sandboxes or climbing frames, and decrease exposure to hazardous UV-light (Boldemann et al. 2006, 2011).

The results from the kernel density maps (Fig. 3) and estimations of continuous time spent in shaded and sunlit areas (Fig. 4) show that the children on the warmest day avoid sunlit areas (e.g., coolest day, 2022-06-01 vs. warmest day, 2022-06-30). This is also visible in Table 3, where percentage time spent in sunlit areas is higher on cooler days. The fact that radiant load is lower and thermal comfort is enhanced in shaded areas explains why the children avoided the sunlit areas on the warmest day. This finding, likewise, suggests that the 5-year-old children in this study are aware of their thermal status. This is in line with interviews with preschool personnel by Bäcklin et al. (2021) that showed that 4–6-year-old children are more aware of their thermal status compared to younger children. There are, however, other possible influencing factors to why the children avoided the sunlit areas. For example, mobile tables were in sunlit areas on the three first days of measurements, whereas on the remaining days they were placed in shade. Although the tables are not utilized all the time, they are a place where the children rest or drink water. Moreover, the teachers avoided sunlit areas on the warmest day and the children approach the teachers on and off throughout the time when they are outside. Even though the children are aware of their thermal status, mobile furniture and equipment and teachers should be in shaded areas on hot days to decrease the risk of exposure to excessive heat.

The use of GPS-trackers on young children is not new. Clevenger et al. (2020) utilized GPS-trackers and accelerometers on 2–5-year-olds to analyze hotspots of physical activity in preschool playgrounds. Their fieldwork was conducted on warm summer days (26–29 °C) although hotspots were not analyzed in relation to weather conditions, which they specified as one limitation to their study. For example, they mention that it is possible that preschoolers by the end of an outdoor session potentially have a cold spot in shade on warm days. Our results suggest that sunlit areas are avoided on warm days. Although hotspots of physical activity are out of the scope in our study, the methodology applied here can be used to investigate its relation to weather conditions, for example, by analyzing hotspots of high and low pulse or number of steps.

The declining physical activity depicted by shorter distance moved, fewer steps taken, and a decrease in highest registered pulse is in line with findings by, e.g., Harrison et al. (2017) that showed that physical activity decreased with daily mean *T*_*air*_ above 20 °C. It is evident from previous studies that heat stress is hazardous to health and wellbeing of children (Malmquist et al. 2021; Bäcklin et al. 2021; Bernstein et al. 2022). The results presented here also indicate that warm weather conditions have negative effects on physical activity. Boldemann et al. (2006, 2011) showed that physical activity is higher in preschool yards with abundant space and vegetation. Considering that the investigated preschool yard presented here is one of the coolest among the preschool yards in Gothenburg due to its generous amount of vegetation, it is likely that physical activity is high on warm days in comparison to exposed yards. The ample amount of shading from vegetation could also explain why the difference in metabolic heat production is minor between sunlit and shaded areas in the yard (Fig. 6c). The negative effects of sunlit conditions are dampened as the children can move in and out of shade regularly and with ease (Fig. 4) and physical activity can be upheld for a longer time. Thus, it is possible that the negative influence of warm weather on physical activity is even larger in exposed preschool yards.

Future research should focus on how warm weather influences the pedagogics and learning of the children and if there are any potential correlations between a decrease in physical activity during warm weather events and, e.g., learning and concentration. Another potential research area is on a possible relationship between the sizes of preschool yards, weather, and physical activity. The size of preschool yards in Gothenburg differs substantially, with a median of 2093 m^2^ (Bäcklin et al. 2021). The yard investigated in the study presented here is 1875 m^2^ with generous space, both sunlit and shaded, for the children to move around in. This, however, is not always the case as two thirds of the 440 preschool yards investigated by Bäcklin et al. (2021) are exposed to heat stress.

### Limitations

The Polar Team Pro sensors that were used to monitor the heart rate of the children in this study have previously, to the author’s best knowledge, never been tested on 5-year-olds. Vanos et al. (2017) successfully used the Polar Team Pro for 9–13-year-olds. Furthermore, they were able to evaluate the simulated energy balance with actual thermal sensation of the adolescents from questionnaires. In our study, the children were considered too young to fully understand the concept of thermal sensation and comfort and were therefore not asked about their actual thermal sensation, even though they seemingly are aware of their thermal status. Moreover, COMFA-kid (Cheng and Brown 2020) have not been evaluated on children as young as in our study. Cheng and Brown (2020) evaluated COMFA-kid on 7–12-year-olds, as they perceived answers on actual thermal sensation by 7-year-olds and older as consistent, whereas survey answers by children younger than 7 years old were noticeably inconsistent.

The thresholds that are utilized here are developed for the hot and dry climate of Phoenix, AZ, USA (Harlan et al. 2006). It is probable that thresholds for Swedish conditions are lower, considering that the climate in Gothenburg, Sweden, is colder and acclimatization is a factor in sensitivity to heat (Baccini et al. 2008; Kovats and Hajat 2008). Cheng and Brown (2020) proposed a scale for children where energy balances in the range 40–<80 Wm^−2^ are considered too warm and energy balances above 80 Wm^−2^ are too hot. This scale, on the other hand, is based on one study with 7–12-year-old children in College Station, TX, USA, whereas the scale by Harlan et al. 2006 is based on NOAA’s National Weather Service Heat Index. NOAA’s Heat Index estimated from extensive biometric studies and assumed more robust. Nevertheless, both scales (Harlan et al. (2006) and Cheng and Brown (2020)) are from climates contrasting to that of Gothenburg. Thus, thresholds for Swedish conditions should be addressed in future research.

The study was conducted in only one preschool yard. This preschool yard is one of the better ones in Gothenburg with regard to vegetation and accesses shade while simultaneously having sunlit areas, thus leaving options for the children to spend time in either shade or sun. It would, however, be beneficial to conduct a similar study in preschool yards with less vegetation and accesses shade to investigate the potential effect of warm weather on their children’s activity and wellbeing. A larger sample group would also be beneficial.

## Conclusions

Physical activity, depicted by distance moved, steps taken, and highest registered pulse, of the preschoolers decreases with warmer weather. Furthermore, it is evident from GPS-tracks that the children, on the warmest day, avoid sunlit areas and spend more time in shaded areas and that percentage of time spent in sunlit areas is larger on cooler days. This indicates that the 5-year-old children observed here are aware of their thermal status. The results on the simulated energy balance of the children are in line with previous research showing that shading is crucial to mitigate heat stress. These results emphasize the importance of shaded areas in preschool yards where children can seek relief from excessive heat gain and retain their thermal status at a safe level while simultaneously continuing their active play.

The measurement of the children’s heartbeat, step count, and distance moved was successfully observed with Polar Team Pro. This methodology can be used to analyze hotspots in preschool yards or playgrounds in relation to weather conditions.

### Supplementary information

ESM 1 (DOCX 17 kb)
