# Supplementary material for: The effects of warm weather on children’s outdoor heat stress and physical activity in a preschool yard in Gothenburg, Sweden
Source: Int J Biometeorol. 2023 Sep 19;67(12):1927–40. doi: 10.1007/s00484-023-02551-y (PMC10643434; doi:10.1007/s00484-023-02551-y)
Supplement: Supplementary file 1 — (DOCX 17 kb) [file 484_2023_2551_MOESM1_ESM.docx]

Supplementary

This short supplementary methods section describes the workflow for calculations of the energy balance in the presented study.

**Observed meteorological data**

We used a stationary weather station, where instruments were mounted on a tripod ~1.1 m above ground. The instruments are given in Table S1. Meteorological data was measured every 5 seconds and averaged over 5 minutes.

Table S1. Table with the instruments used in the study, their variables and units and corresponding reference.

| Instrument | Variable | Unit | Reference |
| --- | --- | --- | --- |
| Vaisala Weather Transmitter WXT 520 | Air temperature | °C | Vaisala (2012) |
|  | Relative humidity | % |  |
|  | Wind speed | m/s |  |
|  | Wind direction | ° |  |
| Delta-T SPN1 Sunshine Pyranometer | Global shortwave radiation | Wm^-2^ | Wood (2019) |
|  | Diffuse shortwave radiation | Wm^-2^ |  |

**Simulations in SOLWEIG**

The measured weather conditions were used as input into the SOLWEIG model (Lindberg et al., 2008). SOLWEIG is capable of calculating shadows and radiation conditions (short- and longwave radiation) from all cardinal directions in all cells. Moreover, a digital surface model with information on building locations, elevations and ground elevation, a canopy digital surface model with information on locations of trees, their canopies and elevation, and a ground cover raster with information on albedo and emissivity of different surfaces were used as input. We used 0.5 m spatial resolution on the input raster data. The temporal resolution was based on the observed meteorological data, i.e., 5-minute average values.

Table S2. Table showing the input data to the SOLWEIG model.

| Input data | Information | Type |
| --- | --- | --- |
| Meteorological data | Air temperature | Meteorological text file |
| Meteorological data | Relative humidity | Meteorological text file |
| Meteorological data | Direct shortwave radiation | Meteorological text file |
| Meteorological data | Diffuse shortwave radiation | Meteorological text file |
| Digital Surface Model | LiDAR derived building and ground elevation | Raster data |
| Digital Elevation Model | LiDAR derived ground elevation | Raster data |
| Canopy Digital Surface Model | LiDAR derived tree elevation | Raster data |
| Ground cover | Albedo and emissivity of the ground | Raster data |

**Child physical activity**

Child physical activity was observed with Polar Team Pro sensors (Polar, Kempele, Finland). The Polar Team Pro sensors measure heart rate (bpm), speed (m/s), distance (m), steps (per minute) and their locations with GPS-tracks (lat and lon). The Polar Team Pro recorded every 0.1 seconds and values were averaged every 1 second. Locations on every full second were used for lat and lon, i.e., not averaged.

**Estimations of the energy balance using COMFA-kid**

We used the COMFA-kid (Cheng & Brown, 2020) to estimate the energy balance of the children. Meteorological conditions were measured with the weather station and used as input into the SOLWEIG model. SOLWEIG can calculate shadows for each time step based on the position of the sun on the sky vault and the elevation of buildings and trees. COMFA-kid uses input incoming short- and longwave radiation, as well as reflected shortwave radiation from ground surfaces and emitted longwave radiation from ground surfaces. These inputs were calculated in SOLWEIG and used as input into the COMFA-kid model. SOLWEIG has no influence on air temperature, relative humidity and wind speed. Therefore, these values are taken directly from the weather station and used as input into the COMFA-kid model. As mentioned, the Polar Team Pro recorded every 1 second. These values (GPS-tracks and heart rate) were combined with closest 5-minute meteorological data and output from SOLWEIG.

The metabolic equivalent task (MET) used as input into the COMFA-kid model was estimated from heart rate measured with the Polar Team Pro. This was done following the methods by Strath et al. (2000).

**Kernel density maps**

Maps showing which areas of the preschool yard that the children visited and how often they frequented the areas were created using the Heatmap (Kernel Density Estimation) tool in QGIS. The Kernel Density Estimation was run with 0.5 m search radius, quartic kernel shape and raw output scaling.

**Continuous time spent in sunlit and shaded areas respectively**

The hist plot showing average continuous time spent in either sunlit or shaded areas per child was created by counting continuous minutes spent in respective area per child and day. The values were averaged as the number of children attending varied between days and thus represented average time per child.
